# Supplemental effects of *Haematococcus pluvialis* in a low-fish meal diet for *Litopenaeus vannamei* at varying temperatures: growth performance, innate immunity and gut bacterial community

**DOI:** 10.3389/fimmu.2024.1501753

**Published:** 2024-12-10

**Authors:** Sihan Lin, Mengdie Chen, Xuanqi Chen, Yanmei Li, Yafeng Liu, Peinan Zhang, Xiangyan Hou, Beiping Tan, Jin Niu

**Affiliations:** ^1^ State Key Laboratory of Biocontrol, Guangdong Provincial Key Laboratory for Aquatic Economic Animals and Southern Marine Science and Engineering Guangdong Laboratory (Zhuhai), School of Life Sciences, Sun Yat-sen University, Guangzhou, China; ^2^ Algae Health Science Co., Ltd., Kunming, China; ^3^ Laboratory of Aquatic Animal Nutrition and Feed, Fisheries College, Guangdong Ocean University, Zhanjiang, China

**Keywords:** *Litopenaeus vannamei*, *Haematococcus Pluvialis*, temperature, growth performance, innate immunity

## Abstract

This study examined the effects of *Haematococcus pluvialis* on the growth performance, innate immunity, and gut microbiota of *Litopenaeus vannamei* under different water temperature conditions. Feeding regimens included a 20% fishmeal diet (control), a low-fish meal (LFM) diet with 10% fishmeal and an LFM diet supplemented with 0.03% *H. pluvialis*. These diets were administered to six groups of *L. vannamei* at normal (30°C) (NT) and low (20°C) (LT) temperatures (NT_C, NT_LFM, NT_LFM_HP, LT_C, LT_LFM, and LT_LFM_HP) over 8 weeks. The weight gain rate of *L. vannamei* in group NT_LFM_HP was significantly higher compared to group NT_LFM. Astaxanthin levels and body pigmentation intensity in *L. vannamei* were significantly increased in the NT_LFM_HP and LT_LFM_HP groups. Moreover, hepatopancreatic antioxidant capacities, such as superoxide dismutase (SOD) activity and total antioxidant capacity (T-AOC), were lower in normal-temperature groups compared to the low-temperature groups. Nevertheless, antioxidant capacity was significantly higher in both the NT_LFM_HP and LT_LFM_HP groups compared to the control group. Meanwhile, the expression levels of antioxidants were significantly higher at lower temperatures compared to higher temperatures, with the NT_LFM_HP and LT_LFM_HP groups exhibiting the highest expression levels. Additionally, the mRNA levels of genes associated with the Toll and IMD pathways indicated immunoregulatory effects in the organism. The expression levels of immune genes were significantly higher at lower temperatures, especially in the NT_LFM_HP and LT_LFM_HP groups compared to the control groups. Notably, significant differences in gut microbial composition were observed in the NT_LFM_HP group compared to other groups, with variations influenced by temperature and fishmeal content. Specifically, Vibrionaceae abundance was significantly lower in the LT_LFM_HP group compared to the control group. The results also revealed that the abundance of Actinomarinales was significantly higher in low-temperature groups, with the LT_LFM_HP group displaying the greatest increase. Overall, these findings suggest that *L. vannamei* may be susceptible to reduced fishmeal levels, potentially impacting growth and immune function. Furthermore, *H. pluvialis* supplementation may assist *L. vannamei* in acclimating to prolonged low-temperature conditions.

## 1 Introduction

As is well documented, *Litopenaeus vannamei* (*L. vannamei*) is an economically important aquaculture species worldwide. Fishmeal is considered the optimal source of protein in shrimp feed owing to its palatability, balanced nutritional composition, and ease of digestion and absorption. However, the price of fishmeal has increased in recent years. To cope with this change, it is essential to develop suitable protein sources to replace fishmeal. In low fishmeal diets, functional additives are introduced to ensure the growth performance and health of aquatic animals. Meanwhile, aquaculture has suffered from environmental destruction over the past decades (1). The extensive administration of antibiotics to enhance disease resistance in shrimp (2) resulted in numerous adverse effects (3). Consequently, the use of pure natural functional additives has become a trend. Shrimp body pigmentation influences consumer preference. However, shrimp are unable to synthesize astaxanthin, and earlier studies have reported that shrimp can use astaxanthin from their diet to optimize body pigmentation. Thus, natural sources of astaxanthin, such as *Haematococcus pluviali* (*H. pluvialis*), have emerged as prominent functional additives. Known to be the richest natural source of astaxanthin, *H. pluvialis* contains astaxanthin levels ranging from 1.0% to 7.0% (4). Therefore, the introduction of astaxanthin to feed can improve growth performance in *L. vannamei* (5), mitigate hepatopancreatic damage in white shrimp (6), improve immune levels in crayfish (7), and improve body color in Japanese shrimp (8).

The growth, health, and immunity of aquatic organisms are considerably impacted by water temperature. For instance, *L. vannamei* is a poikilothermic species whose growth, health, and immunity are regulated by fluctuations in environmental temperature (9). Indeed, temperature stress has been documented to significantly impact shrimp growth and immunity (10, 11), thereby promoting susceptibility to bacterial, fungal, viral, and parasitic infections and economic losses (12). The nutritional metabolism and immune function of shrimp are directly affected by the gastrointestinal tract, which plays a vital role in nutrient absorption and disease resistance. Throughout the growth of shrimp, the structure of the gut flora significantly changes (13, 14). According to earlier studies, alterations in temperature can affect the gut flora of shrimp, thereby influencing their growth and immune performance (15, 16).

To the best of our knowledge, no studies have investigated the effects of *H. pluvialis* on *L. vannamei* under different water temperatures. Given that other aquatic organisms are influenced by the multiple regulatory mechanisms of *H. pluvialis*, this study aimed to investigate the effects of *H. pluvialis* on the growth performance, antioxidant capacity, immune activity, and gut microbiology of *L. vannamei* at distinct water temperatures.

## 2 Materials and methods

### 2.1 Experimental diets

Prior to diet preparation, all raw materials were crushed using a grinder and subsequently sieved using a 60-mesh sieve. After sieving, the raw materials were weighed based on the diet formula outlined in **Table 1**. Briefly, the weighed raw materials were thoroughly mixed in a plastic zip-lock bag and then transferred to a commercial mixer for mixing and blending, during which fish oil, soya lecithin, and water were added and mixed. After mixing, the ingredients were extruded into long strips using a twin-screw extruder and subsequently transferred to a pelletizer to produce pellets for the trials. Next, diets were steam-conditioned at 60°C, then removed and air-dried until the moisture content of the diet was reduced to approximately 10%. Following packing into plastic ziplock bags, the diets were stored in a refrigerator at -20°C. Different feeding regimes, specifically the 20% fishmeal group, the 10% fishmeal group, and the 10% fishmeal + 0.03% *H. pluvialis* group, were administered to *L. vannamei* (NT_C, NT_LFM, NT_LFM_HP, LT_C, LT_LFM, and LT_LFM_HP) at 30°C and 20° water temperature environments.

**Table 1:** Ingredients and proximate composition of six experimental diets (g/kg).

Ingredients	C (NT/LT)	LFM (NT/LT)	LFM_HP (NT/LT)
Fish meal ^1^	20	10	10
Decorticated soybean meal ^2^	18	21	21
Peanut meal ^3^	12	12	12
Chicken meal ^5^	7	7	7
Soy protein concentrate ^6^	4	11	11
Wheat flour ^6^	24.86	23.94	24.21
Hermetia illucens meal ^7^	6	6	6
Beer yeast ^8^	2	2	2
Fish oil ^9^	0.5	1.5	1.5
Soybean lecithin ^10^	1	0.8	0.5
Vitamin premix ^11^	0.5	0.5	0.5
Mineral premix ^12^	0.5	0.5	0.5
Choline ^13^	0.2	0.2	0.2
Cholesterol ^14^	0.1	0.1	0.1
Ca(H2PO4)2 ^15^	1.7	1.7	1.7
Lysine ^16^	0.1	0.17	0.17
Vitamin C ^17^	0.1	0.1	0.1
Methionine ^18^	0.2	0.25	0.25
Threonine ^19^	0.23	0.23	0.23
Y2O3 ^20^	0.01	0.01	0.01
Sodium alginate ^21^	1	1	1
*Haematococcus Pluvialis* ^22^	0	0	0.03
Total	100	100	100
Moisture	8.42	8.45	8.37
Crude lipid	7.62	7.66	7.38
Crude protein	40.21	40.22	40.26
Ash	11.26	11.21	11.23

^1^ Fish meal: Guangzhou Chengyi Industrial Group Co., Ltd., China.

^2^ Decorticated soybean meal: Yihai Kerry Jinlongyu Grain and Oil Food Co., Ltd., China.

^3^ Peanut meal: Zhuhai Dehai Biotechnology Co., Ltd., China.

^4^ Chicken meal: Zhuhai Dehai Biotechnology Co., Ltd., China.

^5^ Soy protein concentrate: Kyorin Industry (Shenzhen) Co., Ltd., China.

^6^ Wheat flour: Hebei Jinshahe Noodle Industry Group Co., Ltd., China.

^7^ Hermetia illucens meal: Qinghai Kunjie environmental protection technology Co., Ltd., China.

^8^ Beer yeast: Guangzhou Chengyi Industrial Group Co., Ltd., China.

^9^ Fish oil: Guangzhou Chengyi Industrial Group Co., Ltd., China.

^10^ Soybean lecithin: Guangzhou Chengyi Industrial Group Co., Ltd., China.

^11^ Vitamin premix (kg^−1^ of mixture): vitamin A, 250,000 IU; riboflavin, 750 mg; pyridoxine HCL, 500 mg; cyanocobalamin, 1 mg; thiamin, 500 mg; menadione, 250 mg; folic acid, 125 mg; biotin, 10 mg; a-tocopherol, 3750 mg; myo-inositol, 2500 mg; calcium pantothenate, 1250 mg; nicotinic acid, 2000 mg; vitamin D3, 45,000 IU; vitamin C, 7000 mg. Guangzhou Chengyi Company Ltd., China.

^12^ Mineral premix (kg^−1^ of mixture): Zn, 4000 mg; K, 22,500 mg; I, 200 mg; NaCl, 2.6 g; Cu, 500 mg; Co, 50 mg; FeSO4, 200 mg; Mg, 3000 mg; Se, 10 mg. Guangzhou Chengyi Company Ltd., China.

^13^ Choline: Guangzhou Chengyi Industrial Group Co., Ltd., China.

^14^ Cholesterol: Guangzhou Chengyi Industrial Group Co., Ltd., China.

^15^ Ca(H_2_PO_4_)_2_: Guangzhou Chengyi Industrial Group Co., Ltd., China.

^16^ Lysine: Shanghai Feeel Technology Development Co., Ltd., China.

^17^ Vitamin C: Guangzhou Chengyi Industrial Group Co., Ltd., China.

^18^ Methionine: Shanghai Feeel Technology Development Co., Ltd., China.

^19^ Threonine: Shanghai Feeel Technology Development Co., Ltd., China.

^20^ Y_2_O_3_: Shanghai Haohong scientific Co., Ltd., China.

^21^ Sodium alginate: Nanjing Duly Biotechnology Co., Ltd., China.

^22^ *Haematococcus Pluvialis*: The effective content of astaxanthin is 3%. Algae health Science Co., Ltd., China.

### 2.2 Feeding experiments

In Lingshui Li Autonomous County, *L. vannamei* was used as the test animal for an 8-week culture experiment. Shrimp larvae were temporarily raised on commercial feed for 10 weeks before being used in the culture experiment. A total of 720 shrimp with an average weight of approximately 0.63 g were randomly selected from the temporarily reared shrimp and assigned to 24 cement tanks, each containing 30 shrimp. Sewage pipes were installed at the bottom of each cement tank for effluent discharge, and water pipes were installed in the cement tanks for seawater replenishment. The water was changed uniformly and regularly according to predetermined water quality indices, with 80% of the water replaced at each interval. All seawater used in the aquaculture tanks was sand-filtered, precipitated, sterilized, and filtered prior to use. The cement tanks were fitted with an air tube and air stone to maintain 24-hour continuous aeration.

Feeding rates were initially set at 5% of shrimp body weight and adjusted based on satiation by observing bait residues after feeding. All groups were fed three times daily, and dead shrimp, bait residues, feces, and other bottom waste were aspirated by suction, and dead shrimp were weighed and counted. During the 8-week aquaculture experiment, the temperatures of the water were maintained at 30°C for the normal-temperature groups and 20°C for the low-temperature groups. A chiller and a water recycling system were used to adjust water temperature, which was recorded every 4 hours. Throughout the experiment, natural light cycles were maintained.

### 2.3 Sampling

During the 8-week culture period, 5 g of feces from each cement tank were collected and stored in a refrigerator at -20°C for apparent digestibility analysis. All shrimp were fasted for 24 hours before sampling. Shrimp in each tank was counted and weighed. The body length, weight, and hepatopancreatic weight of five shrimp from each group were measured and recorded. A total of five shrimp were randomly selected from each group and stored at -20°C for whole shrimp crude nutrient analysis. Frozen hepatopancreas samples harvested from four shrimp were analyzed for enzyme activity and gene expression. Intestinal samples were collected from four shrimp and frozen for intestinal flora analysis. For H&E staining and sectioning, hepatopancreas samples collected from two shrimp were used. For comparison, five live shrimp from each group were boiled under identical conditions.

### 2.4 Determination of proximate composition

According to the guidelines of the Association of Official Analytical Chemists (AOAC), moisture, crude protein, and crude lipid content were determined from dried samples. After drying at 105°C, moisture content was determined, following which the samples were crushed. Next, a fully automated Dumas nitrogen tester (N Pro (DT Ar/He Basic), Gerhardt GMBH & CO.KG, Germany) was employed to determine crude protein content from 0.08 g of sample. The crude lipid content was determined using the Soxhlet extraction method on an automatic lipid analyzer (Soxtec System HT6, Tecator, Sweden) with light petroleum reflux extraction on approximately 0.5 g of the sample. Shrimp shell samples were freeze-dried and ground to extract astaxanthin and measure astaxanthin levels. Ionophore atomic emission spectrometry (ICP-AES) was utilized to quantify metal Y-ions in feces, and apparent digestibility was calculated. The methods for extracting astaxanthin in shrimp shells were performed as described in a previous study (50).

### 2.5 Determination of hepatopancreatic enzyme activities

After thawing hepatopancreatic tissues, samples were collected and added to PBS solution for grinding. The levels of hepatopancreatic enzyme activity indices were determined by centrifuging at 4000 rpm for 20 min at 4°C and collecting the supernatant. Superoxide dismutase (SOD), total antioxidant capacity (T-AOC), and lipid oxidation (MDA) levels were measured using kits (Nanjing Jiancheng Bioengineering Institute, Nanjing, China).

### 2.6 Total RNA extraction and real-time quantitative PCR

Real-time quantitative PCR (qRT-PCR) was performed for all gene expression assays in the present study. With the *Evo MMLV* Reverse transcription reagent kit (Accurate Biology, Hunan, China), total hepatopancreatic RNA was extracted using the TRIzol method, and cDNA was synthesized via reverse transcription. Moreover, qRT-PCR was performed on a Roche real-time quantitative PCR system (LightCycler 480 II, Roche Diagnostics, Basel, Switzerland). Conditions for the reaction were as follows: pre-denaturation at 95°C for 40 amplification cycles (denaturation at 95°C for 5 s, annealing at 60°C for 30 s, and extension at 72°C for 30 s). A melting curve was plotted (95°C for 20 s, 60°C for 20 s, followed by continuous maintenance at 95°C), and the samples were cooled to 4°C. Shrimp primers used in this study are presented in **Table 2**. The *β-actin* gene served as an internal reference gene for gene expression analysis, and relative expression levels were calculated using the 2^-ΔΔct^ method.

**Table 2:** Real-time quantitative PCR primers for genes of *L. vannamei*.

Gene	Primer sequence(5’ to 3’)	GenBank No.	Product size (bp)
*β-actin* F	CGAGGTATCCTCACCCTGA	AF300705.2	101
*β-actin* R	CGGAGCTCGTTGTAGAAGG	AF300705.2	
*toll* F	ATACCTCAGCTTCACG GCAG	XM_027356519.1	140
*toll* R	TATTCGTCAGCAGAGC AGGC	XM_027356519.1	
*dorsal* F	AGATGGAATGATAGAATG GGAAGC	XM_027382195.1	127
*dorsal* R	GTACACCTTTATGGGGTT CTCTATCTC	XM_027382195.1	
*crustin* F	GAGGGTCAAGCCTAC TGCTG	AY486426.1	157
*crustin* R	ACTTATCGAGGCCAG CACAC	AY486426.1	
*sod* F	GCCACTTGAACCACA CCATC	DQ005531.1	158
*sod* R	GCCAGAGCCTTTCAC TCCAA	DQ005531.1	
*cat* F	GGGTATTGAGGCTTCC CCTG	AY518322.1	151
*cat* R	GGGGCCATCTCTCTG GTAGT	AY518322.1	
*gpx* F	AGAAGAGTTCGGCGAC AAGC	AY973252.2	126
*gpx* R	TCGAAGTTGTTCCCA GGACG	AY973252.2	
*imd* F	TATACATCCTGCCGT TGCCG	FJ592176.1	174
*imd R*	GTTGTGGATAACGGGGC CAA	FJ592176.1	
*relish* F	ATTCTTCTGCGTTTCAAG GTGT	KM204120.1	203
*relish* R	GAGGTATGGTCAGGGTAT GGTG	KM204120.1	
*lysc* F	TACTGGTGCGGAAGC GACTA	XM_027352840.1	165
*lysc* R	GTAAGCCACCCAGGC AGAATA	XM_027352840.1	

β-actin, beta-actin; sod, superoxide dismutase; cat, catalase; gpx, glutathione peroxidase; imd, immune deficiency; lysc, lysozyme C-like.

### 2.7 Morphological microscopy of hepatopancreatic tissues

Hepatopancreatic samples were initially fixed in a 4% paraformaldehyde solution for 24 hours and subsequently transferred to a 70% ethanol solution. Prior to sectioning, the samples were subjected to graded dehydration, paraffin embedding, and fixation, following which the sections were sliced into approximately 3.0 μm thick sections using a slicer. Afterward, the sections were subjected to hematoxylin and eosin staining and examined and imaged under a Nikon orthostatic microscope (Eclipse Ni-E, Nikon, Japan). Finally, the resulting images were analyzed utilizing NIS-Elements viewer software (National Institutes of Health, Bethesda, USA).

### 2.8 16S rRNA gene sequencing and microbiota analysis

The genomic DNA of intestinal microorganisms in the sample was extracted and analyzed for purity and concentration using 1% agarose gel electrophoresis. The purified genomic DNA was subsequently dispatched to Shanghai Majorbio Bio‐pharm Technology Co., Ltd (51) for further processing.

Paired-end (PE) reads generated from Illumina sequencing were initially aligned based on their overlapping regions. Subsequently, sequence quality was assessed and filtered, followed by sample differentiation for operational taxonomic unit (OTU) clustering and species taxonomy analysis to facilitate the calculation of various diversity indices. OTU-based diversity index analyses and sequencing depth detection were conducted, while taxonomic information enabled statistical analyses of community structure at different taxonomic levels. Data analyses were conducted utilizing the Meggie BioCloud platform (https://cloud.majorbio.com). Specifically, mothur software was employed to compute alpha diversity metrics such as Chao 1 and Shannon index, while the Wilcoxon rank sum test was utilized to assess variations in alpha diversity among groups (52). Intergroup differences in alpha diversity were evaluated using an algorithm based on the Bray-Curtis distance, coupled with PCoA (Principal Coordinate Analysis) analysis to examine the similarities in microbial community structures across samples. Additionally, the PERMANOVA non-parametric test was utilized in conjunction with LEfSe (Linear Discriminant Analysis Effect Size) analysis (LDA>2, *P*<0.05) to assess significant variations in microbial community structure among groups, identifying bacterial taxa with differing abundances from phylum to genus level (53).

### 2.9 Statistical analysis

Parameters were calculated using the following formulae: Initial body weight (IBW, g)=initial total wet weight/initial number of tails; Final body weight (FBW, g)=final total wet weight/final number of tails; Weight gain (WG, %)=100×(final body weight-initial body weight)/initial body weight; Specific growth rate (SGR, %/day)=100×(Ln final mean weight-Ln initial mean weight)/number of days; Food intake (FI, g/shrimp)=total food intake/total number of shrimp; Feed conversion ratio (FCR)=dry diet fed/wet weight gain; Conversion factor (CF, g/cm^3^)=100×wet weight/(body length)^3^; Hepatosomatic intake (HSI, %)=100×hepatopancreas weight/wet weight; Survival rate (SR, %)=100×number of terminal surviving tails/number of initial tails; Apparent Digestibility (AD, %)=100× (Y_2_O_3_ intake-Y_2_O_3_ output)/Y_2_O_3_ intake.

Experimental data were expressed as “mean ± standard error”. Two-way ANOVA with Bonferroni multiple comparisons were used to analyze data across treatment groups at the same temperature. Student’s *t*-test was conducted to analyze data in the same treatment groups at different temperatures. *P* < 0.05 was considered statistically significant.

## 3 Results

### 3.1 Growth performance, feed utilization, and morphometric parameters

Following the conclusion of the 8-week period, the growth performance, feed utilization, and morphometric parameters for the six groups of *L. vannamei* were individually recorded, as detailed in **Table 3**. The weight gain rate and specific growth rate of *L. vannamei* in the NT_C, NT_LFM, and NT_LFM_HP groups were significantly higher compared to the LT_C LT_LFM and LT_LFM_HP groups (*P*<0.05). Likewise, the weight gain rate of *L. vannamei* in group NT_LFM_HP was significantly higher compared to group NT_LFM (*P*<0.05). In contrast, the weight gain rate of *L. vannamei* in group NT_LFM_HP was comparable to that in group NT_C. Interestingly, no significant differences were noted in the survival rates of *L. vannamei* across treatment groups (*P*>0.05). Conversely, variations in feed efficiency and apparent digestibility were observed between the normal and low-temperature groups (*P*<0.05).

**Table 3:** Effect of the addition of *H. pluvialis* at varying water temperatures on growth performance and feed utilisation of *L. vannamei*.

Items	NT_C	NT-LFM	NT_LFM_HP	LT_C	LT_LFM	LT_LFM_HP	NT&LT_C	NT&LT_LFM	NT&LT_LFM_HP
IBW (g)	0.62 ± 0.00	0.62 ± 0.00	0.61 ± 0.00	0.62 ± 0.00	0.62 ± 0.00	0.63 ± 0.00	ns	ns	ns
FBW (g)	17.76 ± 0.47b	15.51 ± 0.66a	17.70 ± 0.77b	11.99 ± 0.37	10.61 ± 0.82	10.56 ± 0.35	***	**	***
FI (g/shrimp)	21.89 ± 0.35	22.60 ± 1.15	21.93 ± 0.56	11.66 ± 0.36	11.74 ± 0.72	11.21 ± 0.36	***	***	***
SR (%)	96.66 ± 2.35	95.83 ± 2.16	98.33 ± 1.66	97.50 ± 1.59	96.66 ± 3.33	99.16 ± 0.83	ns	ns	ns
WG (%)	2751.54 ± 10.41^b^	2369.48 ± 24.86^a^	2762.00 ± 24.13^b^	1811.36 ± 64.39	1590.46 ± 46.27	1582.49 ± 63.92	***	**	***
SGR (%/d)	3.98 ± 0.03	3.81 ± 0.06	3.98 ± 0.05	3.51 ± 0.03	3.35 ± 0.10	3.35 ± 0.04	***	**	***
FCR	1.28 ± 0.02	1.52 ± 0.05	1.28 ± 0.04	1.03 ± 0.04	1.20 ± 0.12	1.13 ± 0.03	**	ns	*
CF (g/cm^3^)	1.04 ± 0.00	0.99 ± 0.00	1.00 ± 0.01	0.99 ± 0.00	0.98 ± 0.01	0.99 ± 0.00	***	ns	ns
HSI (%)	4.21 ± 0.18	4.36 ± 0.13	4.01 ± 0.13	4.95 ± 0.17	4.64 ± 0.10	4.56 ± 0.11	*	ns	*
AD (%)	0.83 ± 0.02	0.67 ± 0.04	0.81 ± 0.02	0.46 ± 0.05	0.43 ± 0.09	0.44 ± 0.04	**	ns	***

Values are expressed as the means ± SEM with 4 replicates (n=4). Different letters indicate significant differences in the different treatment group at same temperatures (*P*<0.05). * indicates significant differences in the same treatment group at different temperatures (* 0.01<*P*<0.05; ** 0.001<*P*<0.01; *** *P*<0.001). ns indicates no significant difference (*P*>0.05).

### 3.2 Muscle proximate composition

The moisture, crude protein, and crude lipid content of *L. vannamei* muscle within each experimental group are outlined in **Table 4**. The findings revealed no significant differences in these parameters across the groups (*P*>0.05).

**Table 4:** Effect of the addition of *H. pluvialis* at varying water temperatures on the muscle composition of *L. vannamei* (% dry weigNT).

Parameters (% dry matter)	NT_C	NT_LFM	NT_LFM_HP	LT_C	LT_LFM	LT_LFM_HP
Moisture	76.59 ± 0.01	74.94 ± 0.01	77.95 ± 0.01	75.17 ± 0.01	75.48 ± 0.01	75.17 ± 0.01
Crude protein	77.03 ± 0.80	76.82 ± 0.45	76.87 ± 0.42	76.20 ± 0.65	75.43 ± 0.59	75.92 ± 0.92
Crude lipid	6.18 ± 0.41	5.14 ± 0.25	5.68 ± 0.44	4.88 ± 0.17	4.62 ± 0.31	4.89 ± 0.21

Values are expressed as the means ± SEM with 4 replicates (n=4).

### 3.3 Astaxanthin content

**Figure 1:**
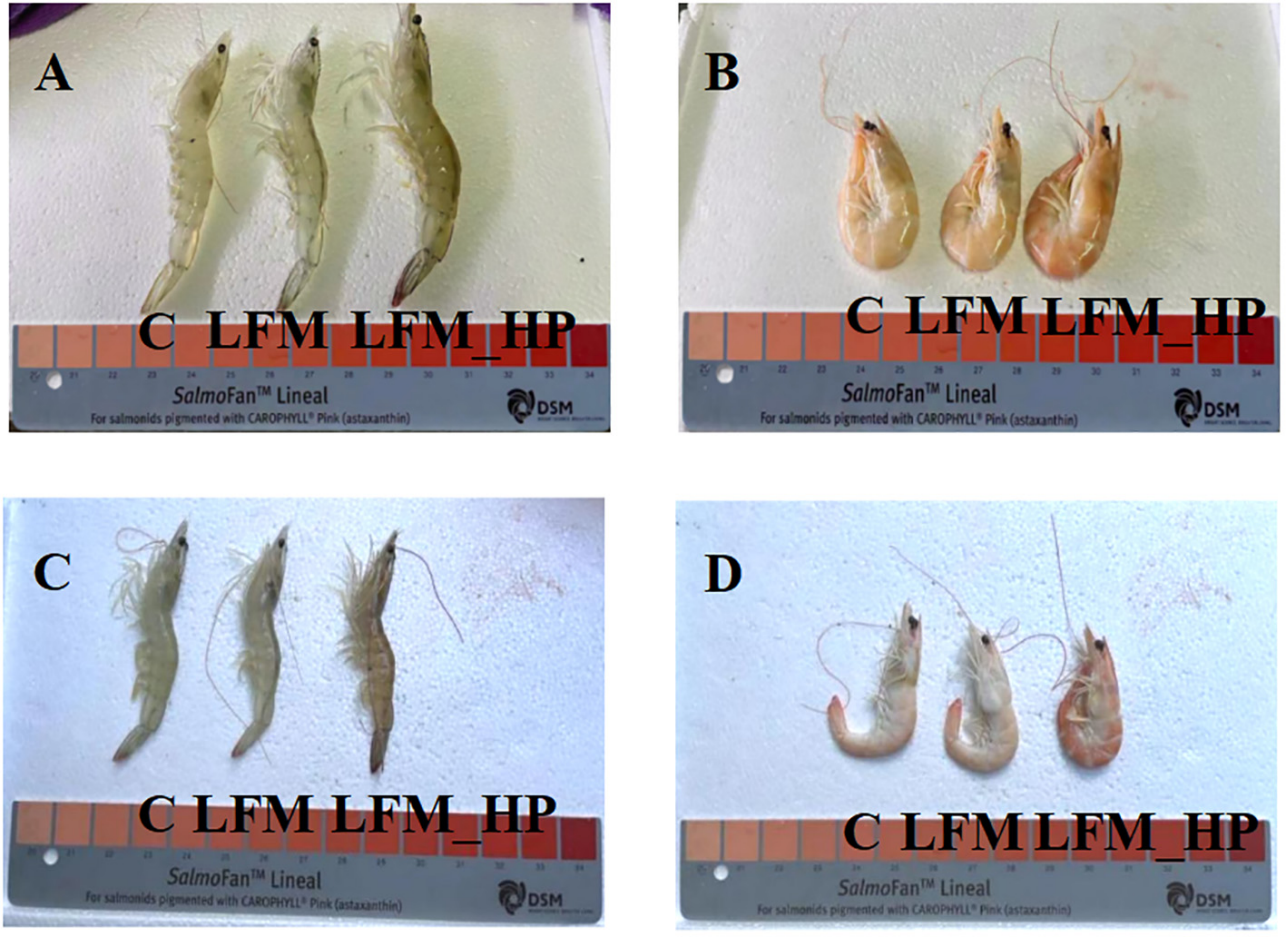
Effect of the addition of *H. pluvialis* at varying water temperatures on the body colour of *L. vannamei*. **(A)** Normal water temperature group of live shrimp; **(B)** Normal water temperature group after cooking shrimp; **(C)** Low water temperature group of live shrimp; **(D)** Low water temperature group after cooking shrimp.

**Figure 2:**
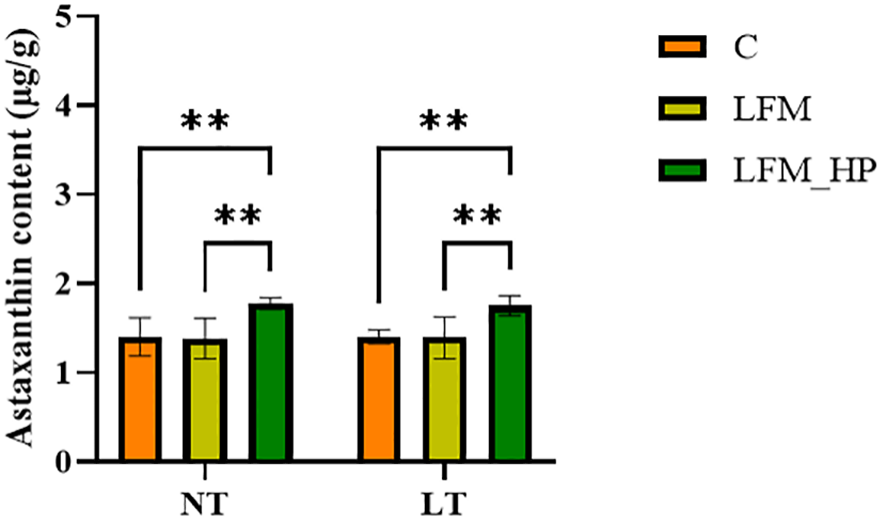
Effect of the addition of *H. pluvialis* at varying water temperatures on the astaxanthin content of shrimp shells of *L. vannamei*. Data represent the means ± SEM (n = 4). * indicates significant differences in the same treatment group at different temperatures (** 0.001<*P*<0.01).

As displayed in **Figure 1**, significant differences were observed in coloration between live and cooked shrimp, with the NT_LFM_HP and LT_LFM_HP groups exhibiting darker pigmentation compared to the NT_LFM and LT_LFM groups. Furthermore, as depicted in **Figure 2**, the astaxanthin content in shrimp shells was highest in the NT_LFM_HP and LT_LFM_HP groups.

### 3.4 Hepatopancreas morphology

**Figure 3:**
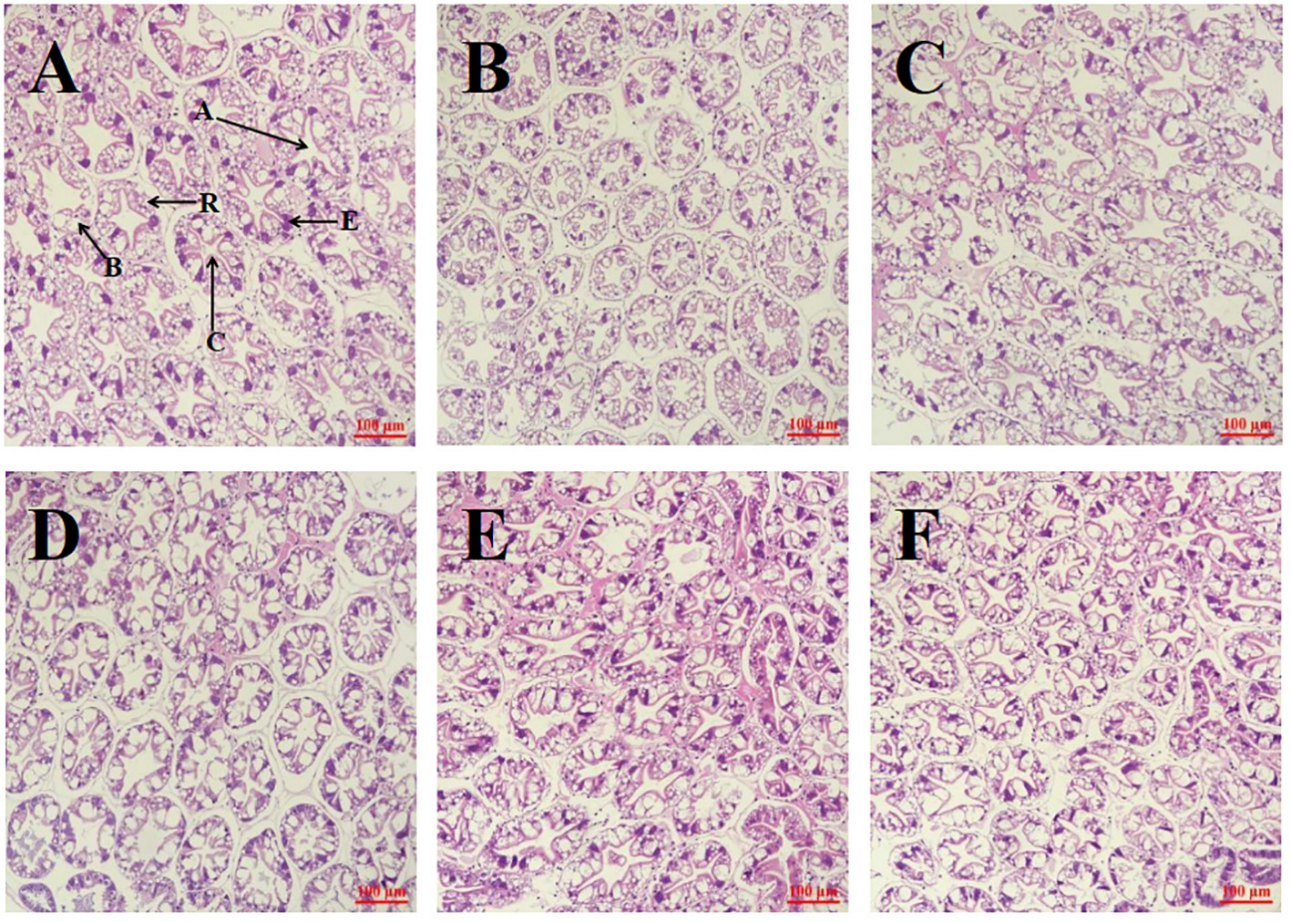
Effect of the addition of *H. pluvialis* at varying water temperatures on the hepatopancreas morphology of *L. vannamei*. Scale bar: 100 μm. Magnification: 20×. **(A)** NT_C group; **(B)** NT_LFM group; **(C)** NT_LFM_HP group; **(D)** LT_C group; **(E)** LT_LFM group; **(F)** LT_LFM_HP group. (A: stellate lumen, C: basement membrane, B: B cell, R: R cell, E: E cell).

**Figure 3** illustrates the comparison of hepatopancreatic tissue sections across groups. The hepatic tubules within the hepatopancreas of groups NT_C, NT_LFM, NT_LFM_HP, LT_C, LT_LFM, and LT_LFM_HP displayed normal morphology, with normal B cells, R cells, and E cells and the absence of lesions.

### 3.5 Hepatopancreas biochemical parameters

#### 3.5.1 Antioxidant enzyme activities

**Figure 4:**
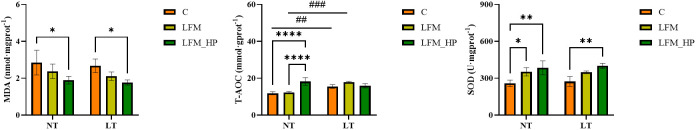
Effect of incorporating *H. pluvialis* at varying water temperatures on the hepatopancreatic antioxidant enzyme activities of *L. vannamei*. MDA, malondialdehyde; T-AOC, total antioxidant capacity; SOD, superoxide dismutase. Data represent the means ± SEM (n = 4).* indicates significant differences in the same treatment group at different temperatures (* 0.01<*P*<0.05; ** 0.001<*P*<0.01; **** *P*<0.0001). # indicates significant differences in the same treatment group at different temperatures (## 0.001<*P*<0.01; ### P<0.001).

**Figure 4** illustrates the impact of *H. pluvialis* supplementation on the hepatopancreatic antioxidant enzyme activities in *L. vannamei* shrimp under varying water temperatures. The findings demonstrated that, following exposure to elevated temperatures, total antioxidant capacity (T-AOC) was significantly higher in the NT_LFM_HP group compared to the NT_C and NT_LFM groups (*P*<0.05). Similarly, SOD activity was significantly higher in the NT_LFM_HP group compared to the NT_C and NT_LFM groups (*P*<0.05). After the inclusion of *H. pluvialis*, MDA levels were significantly lower in the NT_LFM_HP group compared to the NT_C and NT-LF groups (*P*<0.05). Following a decrease in temperature, SOD activity was significantly higher in the LT_C group compared to the LT_LFM_HP group (*P*<0.05). Besides, MDA levels were significantly lower in the LT_LFM_HP groups compared to the LT_C group (*P*<0.05).

#### 3.5.2 Expression of antioxidant-related genes

**Figure 5:**
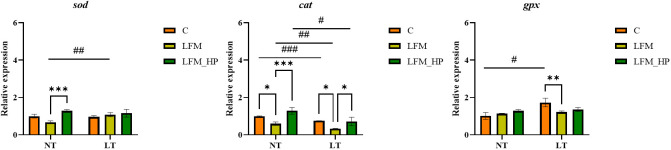
Effect of the addition of *H. pluvialis* at varying water temperatures on the expression of antioxidant genes in the hepatopancreas of *L. vannamei*. Data represent the means ± SEM (n = 4). * indicates significant differences in the same treatment group at different temperatures (* 0.01<*P*<0.05; ** 0.001<*P*<0.01; *** *P*<0.001). # indicates significant differences in the same treatment group at different temperatures (# 0.01<*P*<0.05; ## 0.001<*P*<0.01; ### *P*<0.001).

The gene expression levels of antioxidant-related genes in the hepatopancreas of *L. vannamei* are depicted in **Figure 5**. Significantly lower expression levels of *cat* and *sod* were observed in the NT_LFM and LT_LFM groups compared to the NT_LFM_HP and LT_LFM_HP groups (*P*<0.05). Additionally, the relative expression of *gpx* was higher in the NT_LFM_HP and LT_LFM_HP groups compared to the NT_LFM and LT_LFM groups.

### 3.6 Immune response

**Figure 6:**
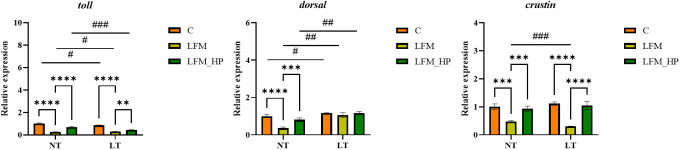
Effect of the addition of *H. pluvialis* at varying water temperatures on the expression of immune genes related to the Toll pathway of *L. vannamei*. Data represent the means ± SEM (n = 4). * indicates significant differences in the same treatment group at different temperatures (** 0.001<*P*<0.01; *** *P*<0.001; **** *P*<0.0001). # indicates significant differences in the same treatment group at different temperatures (# 0.01<*P*<0.05; ## 0.001<*P*<0.01; ### *P*<0.001).

**Figure 7:**
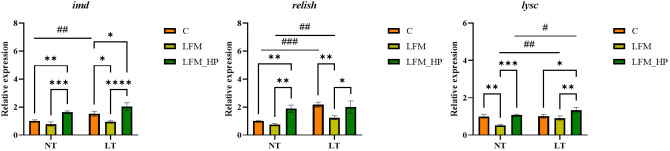
Effect of the addition of *H. pluvialis* at varying water temperatures on the expression of immune genes related to the IMD pathway of *L. vannamei*. Data represent the means ± SEM (n = 4). * indicates significant differences in the same treatment group at different temperatures (* 0.01<*P*<0.05; ** 0.001<*P*<0.01; *** *P*<0.001; **** P<0.0001). # indicates significant differences in the same treatment group at different temperatures (# 0.01<*P*<0.05; ## 0.001<*P*<0.01; ### *P*<0.001).

The Toll and IMD signaling pathways were identified as pivotal elements of the innate immune response in shrimp, as evidenced by the expression of relevant genes depicted in **Figures 6**, **7**.

In the Toll pathway, the relative expression level of *toll* mRNA was significantly higher in the NT_C and LT_C groups compared to the NT_LFM, NT_LFM_HP, LT_LFM, and LT_LFM_HP groups (*P*<0.05). The highest relative expression of *toll* was observed in the NT_C group. At the same time, *dorsal* mRNA expression levels were significantly higher in the LT_LFM_HP group compared to the other groups (*P*<0.05), while *crustin* mRNA expression levels were highest in the LT_C group, and the differences between the groups under different temperature conditions were significant (*P*<0.05).

In the IMD pathway, the relative expression level of *imd* mRNA was significantly higher in the NT_LFM_HP and LT_LFM_HP groups compared to the NT_C, NT_LFM, LT_C, and LT_LFM groups (*P*<0.05). Additionally, the relative expression level of *relish* was higher in the LT_LFM_HP group compared to the NT_LFM_HP group. The mRNA expression levels of *lysc* in the LT_LFM_HP group were significantly higher compared to the other five groups (*P*<0.05), with the lowest expression observed in the NT_LFM group. Specifically, the relative expression of *L. vannamei lysc* mRNA in the LT_LFM_HP group was markedly higher compared to the remaining groups (*P*<0.05), with the lowest expression detected in the NT_LFM group.

### 3.7 Gut microbiota analysis

**Figure 8:**
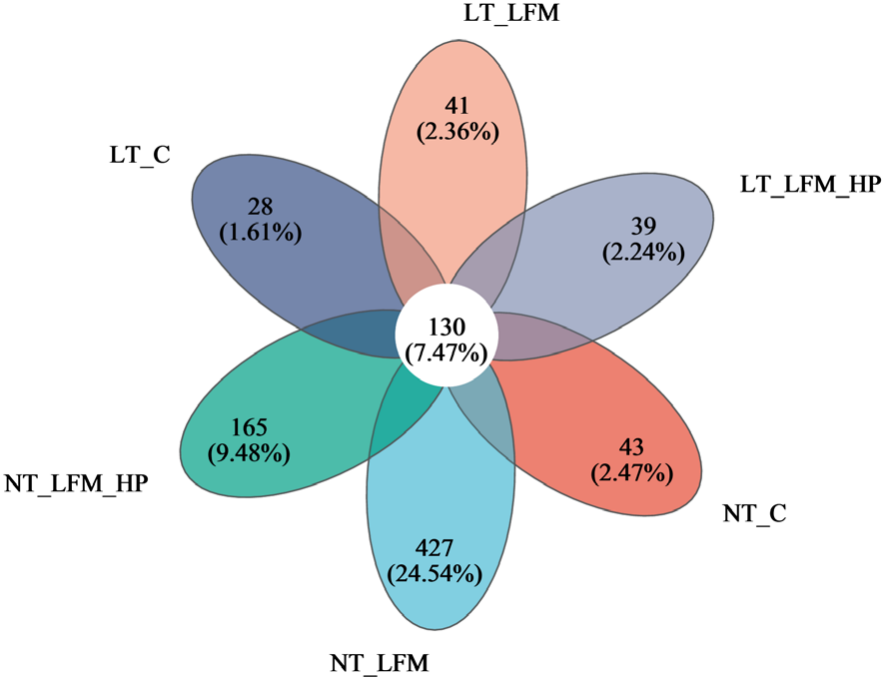
Venn diagram of OTUs Comparison of gut microbiota of the addition of *H. pluvialis* at varying water temperatures in *L. vannamei*.

**Figure 9:**
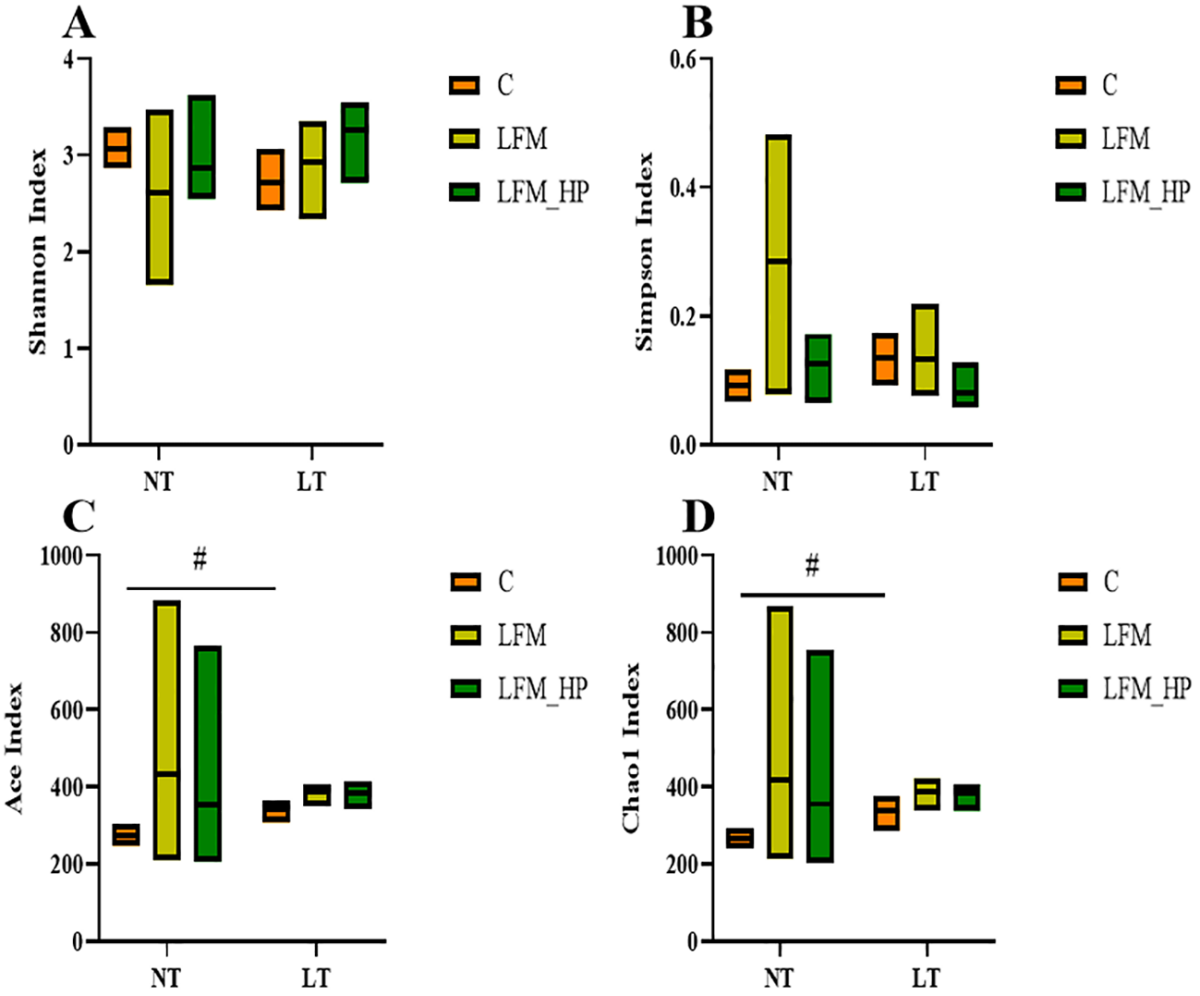
Alpha diversity index statistics of gut microbiota of the addition of (*H*) *pluvialis* at varying water temperatures in *L. vannamei*. Data represent the means ± SEM (n = 4). # indicates significant differences in the same treatment group at different temperatures (*P*<0.05). **(A)** Shannon index; **(B)** Simpson index; **(C)** Ace index; **(D)** Chao1 index.

**Figure 10:**
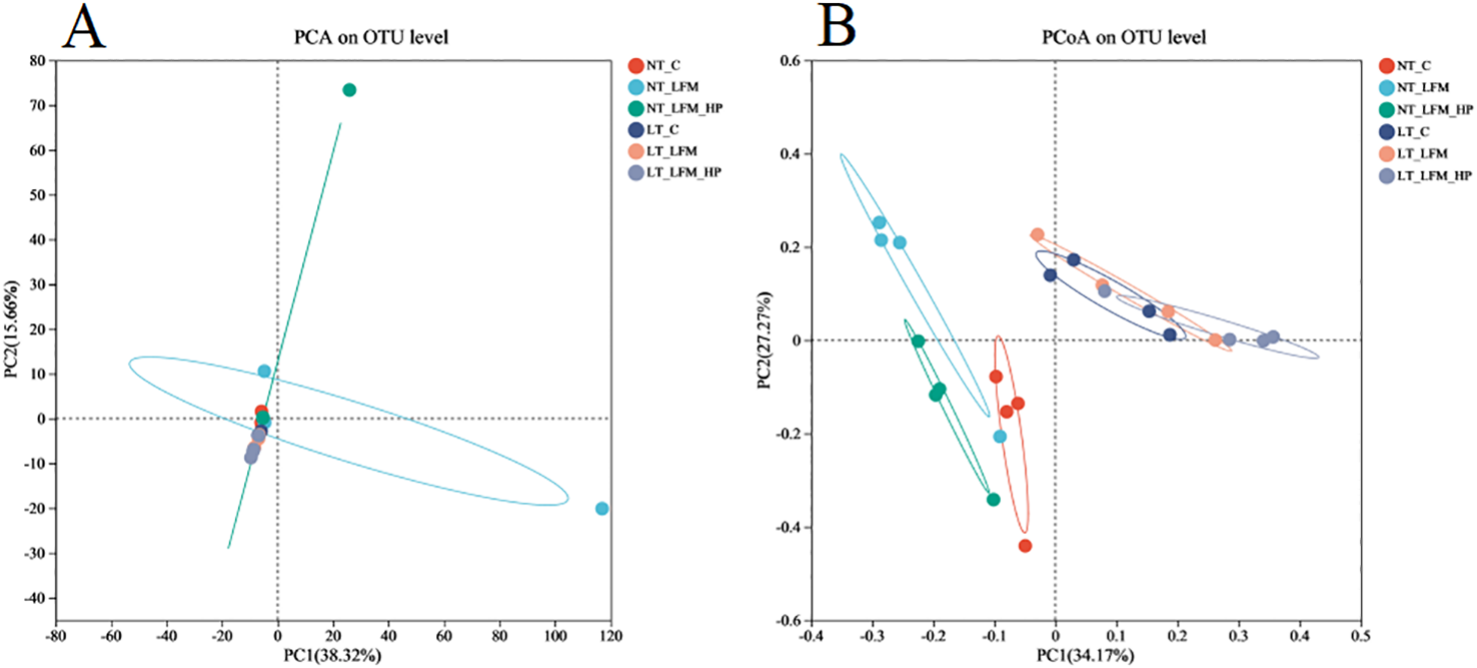
Beta diversity analysis of gut microbiota of the addition of *H. pluvialis* at varying water temperatures in *L. vannamei*. **(A)** PCA on OTU level; **(B)** PCoA on OTU level.

Sequences were grouped into operational taxonomic units (OTUs) at a 97% similarity threshold. The constructed Venn diagram delineated that the NT_C, NT_LFM, NT_LFM_HP, LT_C, LT_LFM, and LT_LFM_HP groups contained 8, 52, 81, 8, 5, and 10 unique Operational Taxonomic Units (OTUs), respectively. As shown in **Figure 8**, significant differences were noted in the microbial community structure due to differences in water temperature and the presence of *H. pluvialis*. **Figure 9** illustrates the analysis of alpha diversity within the shrimp gut microbiome, as determined by sequencing results of the Ace, Chao1, Shannon, and Simpson diversity indices. The findings signaled that the coverage index for each treatment group exceeded 0.998. The beta diversity analysis visualized through PCoA plots (**Figure 10**) demonstrated the impact of varying water temperatures (normal and low) on variations in the intestinal microbiota of *L. vannamei* following exposure to *H. pluvialis*. As anticipated, the composition of the NT_LFM_HP group was significantly different compared to the other groups (*P*<0.05).

**Figure 11:**
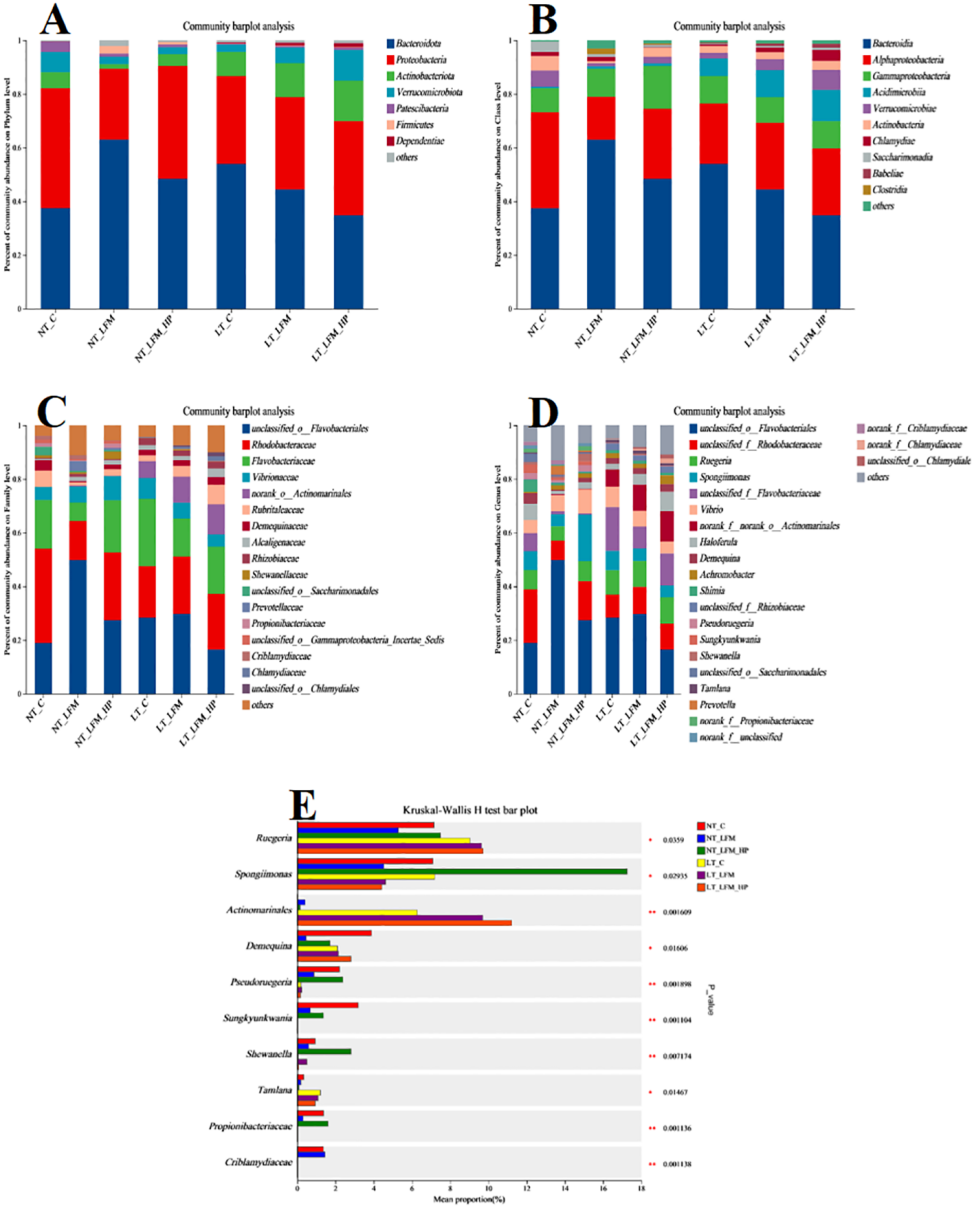
Relative abundance of bacterial community at **(A)** phylum, **(B)** class, **(C)** family, and **(D)** genus levels of gut microbiota of the addition of *H. pluvialis* at varying water temperatures in *L. vannamei*. **(E)** The significant difference among different treatments at the genus level.

**Figure 11** illustrates the distribution of gut bacteria across treatment groups, categorized at the phylum, class, family, and genus levels. Additionally, the relative abundance of species was depicted at each taxonomic level. **Figure 11A** displays the relative abundance of gut bacterial phyla, primarily composed of Bacteroidota, Proteobacteria, Actinobacteriota, and Verrucomicrobia. **Figure 11B** displays the relative abundance of gut bacterial classes in *L. vannamei*, with major bacteria identified as Bacteroidia, Alphaproteobacteria, Gammaproteobacteria, Acidimicrobiia, Verrucomicrobia, and Actinobacteria. **Figure 11C** displays the relative abundance of gut bacterial families, predominantly composed of Flavobacteraceae, Rhodobacteraceae, Vibrionaceae, and Actinomarinales. **Figure 11D** displays the relative abundance of gut bacterial genera, including Flavobacteriales, Rhodobacteraceae, Ruegeria, Spongiimonas, Vibrio, Actinomarinales, and Haloferula.

## 4 Discussion

*L. vannamei* is considered a significant economically cultivated species (17). The growth, development, and metabolic performance of aquatic organisms are influenced by various environmental factors, with water temperature playing a critical role (18). Shrimp, as poikilothermic organisms, exhibit variations in body temperature in response to environmental conditions, which in turn impacts their metabolism and physiological regulatory mechanisms (19). Of note, nutrition, feeding, and feed utilization play crucial roles in commercial aquaculture due to the significant cost associated with feed (20). Consequently, microalgae have garnered considerable attention as a highly nutritious functional green additive in aquafeeds (21). Indeed, incorporating microalgae into aquafeeds has the potential to partially substitute fishmeal and enhance growth, performance, and immunity (22–25).

In the present study, the weight gain rate and specific growth rate of shrimp in the NT_C, NT_LFM, and NT_LFM_HP groups were significantly higher compared to the LT_C, LT_LFM, and LT_LFM_HP groups. Moreover, the weight gain rate of shrimp in the NT_LFM_HP group was higher than that of the NT_LFM group, with no significant difference observed between NT_LFM_HP and NT_C groups, suggesting that both temperature and *H. pluvialis* supplementation influenced shrimp feed intake and subsequently impacted their weight gain and specific growth rates, consistent with the findings of prior investigations indicating that *L. vannamei* exhibited enhanced growth rates within a specific temperature range (26). Noteworthily, no significant differences were noted in the survival rates of *L. vannamei* across treatment groups, whereas variations in feed conversion ratios were observed between the normal- and low-temperature treatment cohorts. In line with the findings of previous studies, at a temperature of 20°C, both the feeding and growth levels of *L. vannamei* were diminished (27). The inclusion of *H. pluvialis* did not yield statistically significant changes in the body composition and hepatopancreas of *L. vannamei* across the experimental groups. Additionally, the morphology of the hepatopancreas and hepatic ducts remained normal in all treatment groups, with B, R, and E cells displaying typical morphology and the absence of evident lesions. Overall, *L. vannamei* in the normal-temperature environment with *H. pluvialis* supplemtation exhibited favorable growth and physiological outcomes. Comparable findings have been documented in studies involving *Pseudosciaena crocea* (*P. crocea*) (28) and *Trachinotus ovatus* (*T. ovatus*) (29).

Body color is a significant factor in evaluating the quality of crustaceans. *H. pluvialis*, a microalgae known for its high astaxanthin content, has garnered interest in meeting the demand for natural pigments in aquaculture (30). Astaxanthin concentrations and body pigmentation intensity in L. vannamei were significantly higher in the NT_LFM_HP and LT_LFM_HP groups, consistent with similar observations in *L. vannamei* and *Marsupenaeus japonicus* (*M. japonicus*) (31, 32).

This study also aimed to examine the antioxidant properties of *H. pluvialis* at varying water temperatures in *L. vannamei*. Carotenoids, non-enzymatic compounds within the antioxidant system, interact with reactive oxygen species (ROS) to mitigate oxidative damage in tissues (33). Superoxide dismutase (SOD) and catalase (CAT) are integral components of an organism’s antioxidant system. Collectively, they play essential roles in scavenging reactive oxygen species, alleviating oxidative stress, modulating the levels of reactive oxygen species, and preserving redox homeostasis (34, 35). In the current investigation, the incorporation of *H. pluvialis* in feeding groups (NT_LFM_HP, LT_LFM_HP) reduced MDA levels, indicative of diminished lipid peroxidation and heightened antioxidant potential, thereby enhancing the capacity to eliminate lipid hydroperoxides in shrimp. Additionally, T-AOC and SOD levels were increased, indicating that the inclusion of *H. pluvialis* may significantly enhance the ability of shrimp to counteract oxygen-free radicals and promote antioxidant defenses. A study investigating *Penaeus monodon* (*P. monodon*) concluded that the inclusion of astaxanthin could enhance antioxidant enzyme activity (36). Analysis of mRNA expression levels of antioxidant-related genes in *L. vannamei* revealed up-regulation of the *cat*, *sod*, and *gpx* genes in groups fed *H. pluvialis* (NT_LFM_HP, LT_LFM_HP), indicating that *H. pluvialis* supplementation may mitigate the decrease in antioxidant capacity caused by low fishmeal diets. Several studies have established that incorporating *H. pluvialis* into the diet could up-regulate the expression levels of antioxidant genes (5, 37, 38), leading to increased SOD enzyme activity in *L. vannamei* cultured at low temperatures compared to those raised at normal temperatures, indicating that prolonged exposure to low temperatures in aquaculture may mitigate oxidative stress and improve antioxidant capacity. The majority of recent studies explored changes in temperature in aquaculture experiments and temperature stress and did not focus on long-term low-temperature aquaculture. Therefore, we theorize that long-term low-temperature aquaculture may enhance the organism’s antioxidant capacity.

In the context of innate immunity in invertebrates, various signaling pathways are activated to regulate immunity through *in vivo* signaling. The Toll and IMD signaling pathways have been extensively investigated in relation to the development of the innate immune system in crustaceans (39). This study further investigated the mRNA levels of genes involved in the immune pathway, specifically focusing on the Toll pathway. The relative expression levels of the *toll*, *dorsal*, and *crustin* genes were significantly higher in the *H. pluvialis* supplementation group compared to the control group. In the IMD signaling pathway, the expression levels of *imd*, *relish*, and *lysc* were elevated in the *H. pluvialis* supplementation group. Similarly, in the IMD immune pathway, the relative mRNA expression levels of *imd*, *relish*, and *lysc* were up-regulated, indicating that exposure to *H. pluvialis* at varying temperatures may attenuate the impact of low fishmeal diets on shrimp immunocompetence. Additionally, in crayfish, the administration of astaxanthin modulated non-specific immunity levels following exogenous challenges (40). Studies have demonstrated that the inclusion of astaxanthin in the diet of *M. japonicus* boosted the immune response (41). Additionally, research indicated that *H. pluvialis* may contribute to the activation of the phenoloxidase cascade, eventually leading to enhanced immunity (42, 43). In this study, the impact of varying water temperatures on the immune system was examined. Our findings indicated that in the Toll signaling immune pathway, the normal water temperature group exhibited lower immune function compared to the low water temperature group. Additionally, in the IMD signaling immune pathway, the low water temperature group displayed significantly higher relative mRNA expression levels of the *imd*, *relish*, and *lysc* genes compared to the normal water temperature group. We postulate that shrimp cultured in a prolonged low-temperature environment may regulate their own immune system, thereby sustaining stable life and health. Moreover, we theorize that under extended low-temperature conditions, shrimp may autonomously modulate its immune response to maintain consistent vital functions.

The growth and health of organisms are influenced by gut microbial communities (44), while water temperature, a critical environmental factor in aquaculture, impacts the compositional structure and dynamic balance of these communities. This study investigated the gut microbiota of *L. vannamei* and identified significant differences between the NT_LFM_HP group and the remaining groups. The analysis of sample complexity revealed a significant difference in the number of operational taxonomic units (OTUs) between the NT_LFM, NT_LFM_HP, and other groups, suggesting that variations in temperature and the presence of *H. pluvialis* may impact the composition of gut microbiota, leading to distinct microbial communities. Beta diversity analysis unveiled that the gut sample points of the NT_LFM_HP group exhibited greater dissimilarity from the sample points of the other groups, with the microbial communities in the NT_LFM_HP group significantly differing from those in the other groups. This observation suggested that *H. pluvialis* influenced the relative abundance of dominant operational taxonomic units (OTUs). The relative abundance plot of the gut flora composition of *L. vannamei* indicated that Proteobacteria, Actinobacteria, and Bacteroidetes were the predominant gut microbial species, consistent with the findings of previous studies (45–47). The findings also indicated that reducing fishmeal content (NT_LFM, LT_LFM) resulted in a higher abundance of Flavobacteraceae. Importantly, *H. pluvialis* supplementation (NT_LFM_HP, LT_LFM_HP) promoted this increase, suggesting that the presence of *H. pluvialis* alleviated the detrimental impact of harmful microorganisms such as Flavobacterium. It is worthwhile emphasizing that the results uncovered significant differences in the abundance of Vibrionaceae and Actinomarinales between low- and normal-temperature environments, with Vibrionaceae being more abundant in normal-temperature environments and Actinomarinales being more abundant in low-temperature environments. This suggested that Actinomarinales may play a decisive role in enhancing shrimp immunity by resisting bacterial invasion and improving immune parameters (48, 49). Furthermore, the findings of this study suggested that exposure to low temperatures may decrease the abundance of detrimental intestinal flora, potentially enhancing shrimp resistance to pathogens through the modulation of intestinal flora composition. Furthermore, the research indicated that cultivating shrimp in low water temperatures could promote immune responses in non-specific immune assays, thereby supporting their overall health and survival.

## 5 Conclusion

The study incorporated varying water temperatures and fishmeal levels, including a group with 20% fishmeal and another with 10% fishmeal supplemented with *H. pluvialis*. The findings exposed that *L. vannamei* growth and immune response were negatively affected by reducing fishmeal levels, whereas *H. pluvialis* supplementation improved growth, antioxidant capacity, and immune function. Additionally, under long-term low-temperature conditions, *L. vannamei* demonstrated enhanced resistance to external pathogens through immune system modulation. Overall, *H. pluvialis*, as a natural additive, exerted positive effects on aquatic animal nutrition, including promoting growth, improving immunity, and improving biochemical indicators. Taken together, these results collectively highlighted the extensive application potential of *H. pluvialis* in the aquatic feed industry owing to its unique characteristics.

## Data Availability

The original contributions presented in the study are included in the article/supplementary material. Further inquiries can be directed to the corresponding authors.
